# A method of detecting apple leaf diseases based on improved convolutional neural network

**DOI:** 10.1371/journal.pone.0262629

**Published:** 2022-02-01

**Authors:** Jie Di, Qing Li

**Affiliations:** Center for Innovation Management Research of Xinjiang, School of Economics and Management, Xinjiang University, Urumqi, Xinjiang, China; Taipei Medical University, TAIWAN

## Abstract

Apple tree diseases have perplexed orchard farmers for several years. At present, numerous studies have investigated deep learning for fruit and vegetable crop disease detection. Because of the complexity and variety of apple leaf veins and the difficulty in judging similar diseases, a new target detection model of apple leaf diseases DF-Tiny-YOLO, based on deep learning, is proposed to realize faster and more effective automatic detection of apple leaf diseases. Four common apple leaf diseases, including 1,404 images, were selected for data modeling and method evaluation, and made three main improvements. Feature reuse was combined with the DenseNet densely connected network and further realized to reduce the disappearance of the deep gradient, thus strengthening feature propagation and improving detection accuracy. We introduced Resize and Re-organization (Reorg) and conducted convolution kernel compression to reduce the calculation parameters of the model, improve the operating detection speed, and allow feature stacking to achieve feature fusion. The network terminal uses convolution kernels of 1 × 1, 1 × 1, and 3 × 3, in turn, to realize the dimensionality reduction of features and increase network depth without increasing computational complexity, thus further improving the detection accuracy. The results showed that the mean average precision (mAP) and average intersection over union (IoU) of the DF-Tiny-YOLO model were 99.99% and 90.88%, respectively, and the detection speed reached 280 FPS. Compared with the Tiny-YOLO and YOLOv2 network models, the new method proposed in this paper significantly improves the detection performance. It can also detect apple leaf diseases quickly and effectively.

## 1. Introduction

With over 2,000 years of history in China, areas of apple implantation have expanded annually, and 65% of all apples in the world are now produced in China. The traditional apple industry has been modernized with the support of national economic policies, and China has gradually developed into a major power source in the apple industry. Nonetheless, behind the rapid development of the apple implantation industry, disease prevention and control have been important problems that have long perplexed orchard farmers [1], who identify apple diseases by referring to experience, books, the Internet, and consulting professional and technical personnel [2]. However, relying solely on these sources is not conducive to timely and effective identification of diseases, and might even cause other problems associated with subjective judgment. Effective automated detection of apple diseases during production not only promptly monitors the health status of apples but also helps orchard farmers to correctly judge apple diseases. They can then implement timely prevention and control to avoid large-scale diseases, which is crucial for promoting the healthy growth of apples and increasing the economic benefits of orchards [3, 4].

Hundreds of apple diseases disseminate in fruits, leaves, branches, roots, and other areas, but often initially appear in leaves, which are easily observed, collected, and managed. Therefore, they are an important reference for disease identification and effective automated detection of diseases is essential. However, judging differences among diseases is difficult due to the complexity of blade veins [5], resulting in unsatisfactory outcomes of experimental detection methods [6].

Nowadays, based on traditional algorithms, much methods has been made in the detection of apple leaf diseases, artificial neural network algorithms, genetic algorithms [7], support vector machines (SVMs), and a clustering algorithm [8] have applied feature extraction to apple leaves, and Li and He [9] established a BP network model with intelligent recognition that can diagnose apple leaf diseases using low-resolution images. Rong-Ming, Wei [10] proposed a method for extracting apple leaf disease spots based on hyperspectral images. Omrani, Khoshnevisan [11] proposed a calculation based on an artificial neural network (ANN) and SVMs to detect three apple leaf diseases. Shuaibu, Lee [12] applied hyperspectral images to detect marssonina blotch in apples at different stages and used orthogonal subspace projection for feature selection and redundancy reduction which generated better results. Zhang, Zhang [7] used genetic algorithm and feature selection method based on correlation to identify three kinds of apple leaf diseases, with an average recognition rate of 90%. Although traditional methods can also extract a large number of classification features, to select a few optimal features using general feature selection methods is difficult, so the recognition rate of these methods is low and generalization ability is weak.

Since the emergence of target detection technology during the 1960s, many domestic and overseas investigators have focused on the development of new technologies. After Krizhevsky, Sutskever [13] proposed the AlexNet structure at the ImageNet Large-scale Visual Recognition Challenge (ILSVRC) in 2012, target detection based on deep learning has gradually become mainstream [14]. Instead of using manual feature extraction, deep learning adopts hierarchical feature extraction [15], which is more similar to the human visual mechanism and has more accurate detection [16]. Many investigations into target detection based on deep learning have emerged [17], and applications to fruit and vegetable crop disease detection have been continuously proposed. Based on the AlexNet model, Brahimi, Boukhalfa [18] classified nine tomato diseases using more than 10,000 tomato disease images with a simple background in the PlantVillage database, further enhancing the model recognition rate. Priyadharshini, Arivazhagan [19] proposed a deep CNN-based architecture (modified LeNet) for four maize leaf disease classifications, and the trained model achieved 97.89% accuracy. In order to effectively examine the minor disease spots on diseased grape leaves, Zhu, Cheng [20] proposed a detection algorithm based on convolutional neural network and super-resolution image enhancement, the test effect is greatly improved. Zhang, Zhang [21] improved the model structure of the LeNet convolutional neural network to solve the problems of leaf shape, illumination, and image background that easily affect detection using traditional recognition methods; their model recognized six cucumber diseases, with an average recognition ratio of 90.32%. Xue, Huang [22], overcame the difficulty of detecting mango overlap or occlusion and proposed the improved Tiny-YOLO mango detection network combining dense connections, with an accuracy rate of 97.02%. Xiao, Li-ren [23] proposed the feature pyramid networks-single-shot multibox detector (FPN-SSD) model to detect top soybean leaflets on collected images, and automatically detect soybean leaves and classify leaf morphology. The wide application of deep learning to the detection of fruit and vegetable diseases has furthered automatic disease detection by leaps and bounds [24, 25].

Convolutional neural network learning methods are now being applied to classify and identify apple leaf diseases, such as the SDD model, and the R-CNN and YOLO series of algorithms. Many improved schemes have been proposed based on existing algorithms. Jiang, Chen [26] proposed the INAR-SSD (SSD with an Inception module and Rainbow concatenation) model to detect five common apple leaf diseases, with high accuracy (78.80% mAP) in real time. The INAR-SSD has provided feasible real-time detection of apple leaf diseases. Liu, Zhang [27] created a new structure based on the deep convolutional neural network AlexNet to detect apple leaf diseases, thus further improving the effective identification of apple leaf diseases. Based on the DenseNet-121 deep convolutional network, Zhong and Zhao [28] proposed regression, multi-label classification, and focus loss function, to identify apple leaf diseases, with a recognition accuracy rate > 93%, which was better than the traditional multi-classification method based on the cross-entropy loss function. Jan and Ahmad [29] developed an apple pest and disease diagnostic system to predict apple scabs and leaf spots. Entropy, energy, inverse difference moment (IDM), mean, standard deviation (SD), and perimeter, etc., were extracted from apple leaf images and a multi-layer perceptron (MLP) pattern classifier and 11 apple leaf image features to train the model, which had 99.1% diagnostic accuracy. Yu, Son [30] proposed a new method based on a region-of-interest-aware deep convolutional neural network (ROI-aware DCNN) to render deep features more discriminative and increase classification performance for apple leaf disease identification, with an average accuracy of 84.3%. Sun, Xu [31] proposed a lightweight CNN model that can be deployed on mobile devices to detect apple leaf diseases in real time to test five common apple leaf diseases, with a mAP value of 83.12%. Di and Qu [32] applied the Tiny-YOLO model to apple leaf diseases, and the results showed that the model was effective.

There are two main types of target detection in the deep learning era [33]. The first is based on region formation. Representative models are R-CNN [34], Fast R-CNN [35], and Faster R-CNN [36]. One method forms candidate boxes that might contain target areas in images, extracts features from each box, trains classifiers, and achieves the target detection of specific objects in two steps. The other method is based on regression. Typical representative models are SDD [37], YOLO [38], and YOLOv2 [39]. These methods address detection as regression problems; they directly predict the coordinates of the boxes, the confidence level of the objects contained, and the probability of the objects appearing, then realizes the target location and identifies the category of specific objects in one step. The detection accuracy of the region formation-based method is superior. However, a target detection method based on regression is more advantageous in terms of detection speed and is popular in terms of real-time detection and intelligent applications. Yet, most recent research efforts focusing on detecting apple leaf diseases are not enough, and considerable room exists for improving detection accuracy and effectiveness. Therefore, the present study investigated how to optimize methods of apple leaf disease detection. Practical problems such as complexity and variations in the veins of apple leaves and difficulties with disease identification can be overcome by the rapid and effective automated detection of apple leaf diseases. Therefore, we created the DF-Tiny-YOLO model to, an detect apple leaf diseases based on regression with the concepts of DenseNet [40] and F-YOLO [41]. We then optimized the model considering rapid, accurate detection.

## 2. Algorithm principle and DF-Tiny-YOLO network

### 2.1 Algorithm principles

#### 2.1.1 YOLO network model

The core principle of YOLO is the consideration that target detection is a regression problem. In this algorithm, images are divided into *S* × *S* grid cells, where each grid predicts B bounding boxes, and each bounding box contains five predictive values (*x*, *y*, *w*, *h*, *confidence*): *x*, *y* indicates the central coordinate of the prediction bounding box relative to the grid boundary, and *w*, *h* indicates the width and height of the prediction bounding box. *Confidence* has two definitions: one is the possibility that a bounding box contains a target, and the other is the prediction accuracy of the bounding box, expressed as the IoU of the joint between the prediction box and the actual bounding box, defined as:

$$\begin{array}{c} \text{Confidence}=Pr\left(object\right)*{IoU}_{prediction}^{truth} \end{array}$$ (1)

In Eq (1), *Pr*(*object*) is the probability that a target object falls into the grid, which is a standard for measuring the detection accuracy of a specific object in a dataset. If a target object is not in the grid, the *Pr*(*object*) value is 0, and the final value is 0; otherwise, the *Pr*(*object*) value is 1, at this point the final confidence value is ${IoU}_{prediction}^{truth}$. The conditional class probability of each grid (*Pr*(*Class*_*i*_|*object*)) is multiplied by the confidence to obtain the confidence of the specific class of objects contained in the prediction box Eq (2):

$$\begin{array}{c} \text{Pr}\left({\text{Class}}_{i}|\text{object}\right)*Pr\left(object\right)*{IoU}_{prediction}^{truth}=Pr\left(Class_{i}\right)*{IoU}_{prediction}^{truth} \end{array}$$ (2)

On this basis, YOLO selects the appropriate candidate region to predict while detecting the input picture to obtain the optimal position and classification of objects in the candidate region. The YOLO network model comprises 24 convolutions and two full-link layers. Redmon, Divvala [38] applied the YOLO algorithm to various datasets to verify its accuracy. The average YOLO target detection accuracy is about 60%, with a recognition rate up to 45 frames/s.

#### 2.1.2 YOLOv2 network

Although YOLO has been greatly improved in terms of detection speed, its detection accuracy is far from optimal. Object positioning is insufficiently accurate, and its recall rate is relatively low. Thus, YOLOv2 has been improved based on the advantages of the original YOLO, which proposes the new network structure, Darenet-19, which includes 19 convolution and five maximum pooling layers and adopts a series of optimization strategies. In terms of candidate box selection, the K-means clustering method is adopted in YOLOv2 to determine the size, proportion, and number of candidate boxes, and obtain reasonable candidate box selection parameters through balanced clustering results and computational complexity. A pass-through layer has been added to the network structure to connect shallow and deep features, which has adapted YOLOv2 to multi-scale features and improved the detection accuracy of small targets. Multi-scale training was adopted, and a group of new pictures was randomly selected for scale input every 10 rounds of training to enhance detection performance. Moreover, many aspects have been improved. The final test results of the VOC 2007 dataset showed that when the size of the input picture was 228 × 228, the frame rate reached 67 FPS and mAP reached 76.8%. When the input picture resolution was 554 × 554, the mAP of YOLOv2 on VOC 2007 reached 78.6% and the frame rate reached 40 FPS, which satisfied real-time requirements.

#### 2.1.3 Tiny-YOLO network

**Fig 1:**
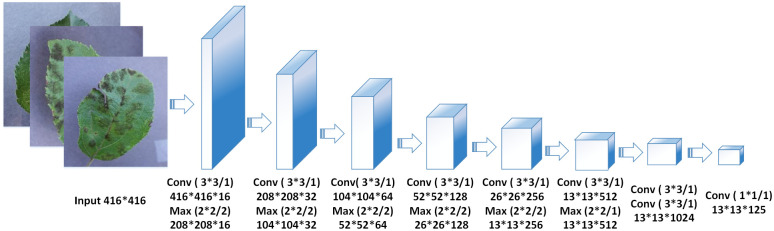
Tiny-YOLO network structure. Tiny-YOLO structure and the network parameter shown in here.

Tiny-YOLO is a miniature “accelerated version” network based on the Darknet-19 structure (Fig 1). This reduces the complexity of the model, and the number of training parameters greatly improves the training speed up to 244 frames/s, which is 1.5-fold the detection speed of YOLOv2. However, noise increased during Tiny-YOLO training due to reduced model depth. Despite progress in speed, target positioning accordingly became inaccurate, resulting in a low average accuracy of 23.7%, compared with YOLOv2, which can achieve 78.6% accuracy at 40 frames/s. The detection accuracy of Tiny-YOLO can still be improved.

#### 2.1.4 Dense connective network

**Fig 2:**
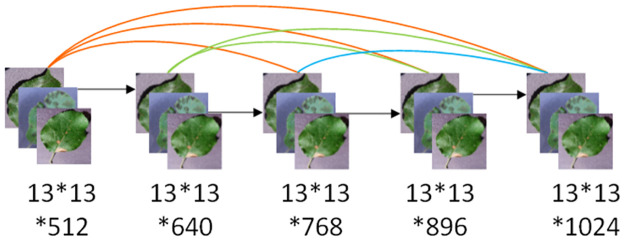
DenseNet network structure.

Huang, Liu [40] connected all layers in the network in pairs so that each layer in the network can receive the characteristics of all layers in front of it as input. This maximized information flow among all layers in the network. This network structure is called DenseNet because of its numerous dense connections (Fig 2).

**Fig 3:**
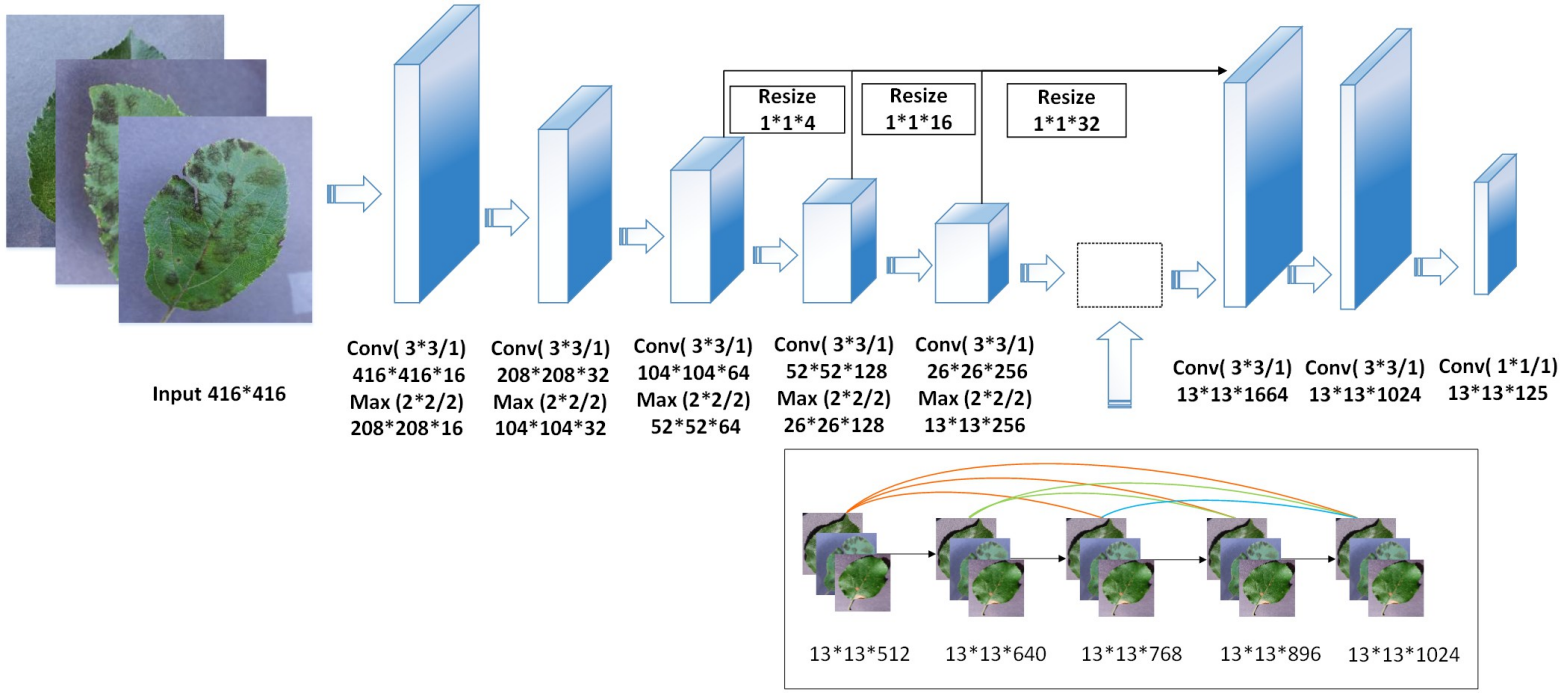
DF-Tiny-YOLO network structure.

DenseNet has the following important characteristics. It alleviates the problem of gradient dissipation during training to some extent. Each layer receives gradient signals from all following layers during reverse transmission, and the gradient near the input layer will not become smaller as the network depth increases. As numerous features are reused, many features can be generated using fewer convolution kernels, so the size of the final model is relatively small. The main purpose of DenseNet was to establish a connection relationship between different layers. In the traditional convolution structure, layer k has k connections, whereas in the DenseNet structure, layer k has *k*(*k* − 1)/2 connections, that is, each layer in the model is connected with the following layer to enhance feature extraction. Fig 3 shows the network structure of DenseNet. Overall, the structure consists mainly of dense blocks and transition layers. The internal feature diagrams of the dense blocks are of the same size, and transition block nodes comprise “BN-Conv-Pool”.

DenseNet encourages feature reuse, which means that the features of each picture can be used, and this reduces disappearance of feature gradient, strengthens the feature propagation of the network model, and effectively inhibits the overfitting problem.

#### 2.1.5 F-YOLO network

Mehta and Ozturk [41] proposed F-YOLO based on Tiny-YOLO. Generally, a deeper and wider network structure, results in a better detection effect, but the corresponding calculation of parameters will also increase, resulting in slow network training. Considering this problem, Mehta et al. used the resize in the YOLOv2 algorithm to compress the convolution layers of the 6th, 8th, and 10th layers by a 1 × 1 convolution kernel, superimpose them on the first 13 × 13 × 1024 convolution layer, and connect them to form a 13 × 13 × 1792 feature diagram. Thereafter, many 1 × 1 convolution layers were added to increase the network depth without increasing the computational complexity, thus producing a “narrow and deep” network. Compared with the original Tiny-YOLO network, the modified network improved the accuracy by 5 mAP and the speed by 20%. On the Pascal dataset, the training parameters of the final model were reduced > 10-fold, and the training speed exceeded 200 FPS compared with the VGG-based target detection model.

### 2.2 DF-Tiny-YOLO network model

The following improvements to the original Tiny-YOLO network structure were made based on existing knowledge to realize the rapid identification and accurate location of apple leaf diseases.

Huang, Liu [40] proposed the concept of a dense connection network. They replaced the 13 × 13 × 512 convolution layer in the Tiny-YOLO structure with a dense module; the 13 × 13 × 128 convolution kernel was used for convolution to output 128 feature diagrams, so that the spliced output of the input and first layers is 13 × 13 × (512 + 128), the output set of the second layer is 13 × 13 × (640 + 128), and so on. The spliced output of the last layer is 13 × 13 × 1024, thus reducing gradient disappearance, and further realizing feature reuse, strengthening feature propagation, and improving detection accuracy.

Resize and reorg were adopted to compress large feature diagrams, reduce network computation, accelerate network training, and improve the speed of detection. Large and small feature diagrams were stacked on the last main convolution layer to achieve feature fusion.

Using 1 × 1, 1 × 1, and 3 × 3 convolution kernels at the network terminals will contribute to reducing the dimension of features and increasing the network depth without increasing computational complexity, thus further improving the detection accuracy.

A DF-Tiny-YOLO network model was constructed with the structure shown in Fig 3 based on these improvements, and the network parameter also shown in here.

## 3. Analysis of experimental process and results

### 3.1 Experimental process

#### 3.1.1 Experimental data

**Fig 4:**
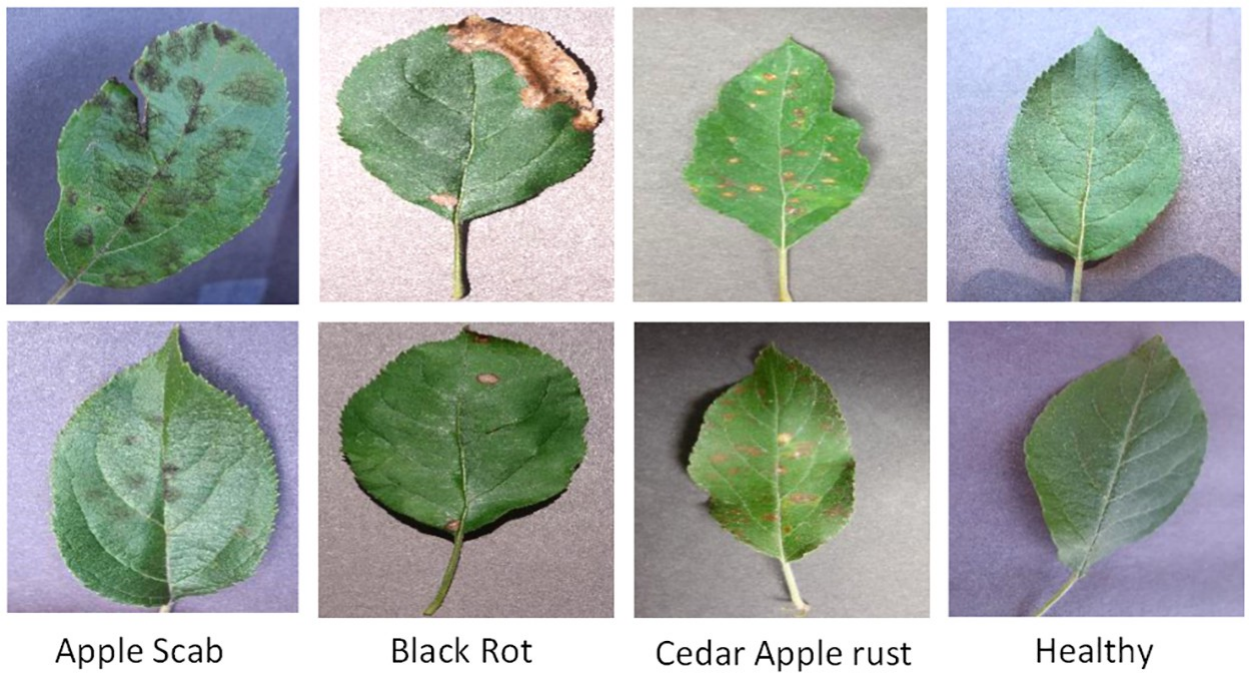
Images of apple leaf diseases. The figure describes the basic situation of four types of leaves.

We randomly selected four clear common apple leaf pictures of the same size in the PlantVillage database as the experimental dataset. The quality of this dataset was ensured by manual screening to avoid the problems of singleness, repeatability, and errors. The data included 300 images of leaves with apple scab, 300 with apple black rot, 275 with apple cedar rust, and 529 healthy leaves, total 1,404 images. Fig 4 describes the basic situation of four types of leaves. The images were randomly divided into training and test sets at a ratio of 80%:20%, the division of data set was shown in Table 1.

**Table 1:** Division of apple leaf data set.

Classes	Raw data set	Training data set	Test data set
**Healthy apple leaves**	529	423	106
**Apple Scab**	300	240	60
**Black Rot**	300	240	60
**Cedar Apple Rust**	275	220	55
**Total**	1404	1123	281

The table shows division of training and test sets.

Many common diseases affect apple leaves. Table 2 summarizes the main characteristics of diseased and healthy leaves with reference to relevant information.

**Table 2:** Comparison of apple leaf disease characteristics.

Disease type	Characteristics
**Healthy apple leaves**	Elliptical or oval, with blunt serrations on leaf edge. Both sides of young leaves are pilose, and front pilose falls off when mature.
**Apple Scab**	Almost round or radial disease spots. Leaves are initially covered with green-brown mildew that later turns black. Diseased spots can cover whole leaves, causing a scorched appearance.
**Black Rot**	Disease spots are initially small and purple-black. These expand into round spots with a yellowish-brown middle with brown purple edges resembling frog eyes.
**Cedar Apple Rust**	Small, oily orange-red disease spots gradually expand and become round orange-yellow and edged in red.

After uniformly labeling the four categories of apple leaf picture data, the leaf samples were manually labeled individually according to the following criteria: label box frames apple leaves in the image regardless of the stem, and the upper, lower, left, and right sides of the label box are close to the outer boundary of the leaves, to minimize errors emerging during sample labeling.

#### 3.1.2 Environment and tools

Table 3 shows the server platform configuration and tools used herein.

**Table 3:** Server platform configuration and tools.

Configuration Item	Value
**CPU**	Intel Core i7–8700K @ 3.70 GHz
**GPU**	Nvidia GeForce GTX 1080 Ti GPU
**Memory**	64GB RAM
**Solid state disk**	Intel SSDSCKKW256H6
**Operating system**	Windows 7 Ultimate 64bit SP1
**Deep learning framework**	TensorFlow 1.3+

#### 3.1.3 Model training

Referring to the training parameters of the original Tiny-YOLO model, the main initial training parameters of the improved DF-Tiny-YOLO model set in this study are shown in the Table 4. Where the learning rate parameter values will be adjusted appropriately during the model training period based on the average loss values.

**Table 4:** The initial parameters of the improved DF-Tiny-YOLO training model.

Parameter name	Parameter value
**batch**	64
**subdivisions**	4
**momenturn**	0.9
**decay**	0.0005
**learning rate**	0.001

**Fig 5:**
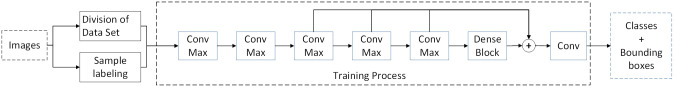
DF-Tiny-YOLO training process. The figure shows the experimental process for the target detection of apple leaf diseases.

Fig 5 shows the experimental process for the target detection of apple leaf diseases. The apple leaf images are first screened, then the entire leaf data set is divided into a training set and a test set, and finally an experimental file containing the corresponding information is generated by annotating the samples. Using the apple leaf training data, the detection training is performed on the original YOLOv2 model, the Tiny-YOLO model, and the modified DF-Tiny-YOLO model, respectively. The DF-Tiny-YOLO network model was generated by improving the Tiny-YOLO network through combining DenseNet with the F-YOLO concept. The model was trained and tested using the training and test datasets, respectively, since the measurement error decreases in many iterations, the network performance is optimized with the correction of parameters. The training ends when the average loss value of the network model decreases to a certain level and the value does not decrease in subsequent iterations, to generate the DF-Tiny-YOLO apple leaf disease detection model. The experimental findings of DF-Tiny-YOLO, YOLOv2, and the original Tiny-YOLO were compared.

#### 3.1.4 Experimental evaluation indexes

The mean average precision (mAP), intersection over union (IoU), recall, and frame per second (FPS) were adopted as the evaluation indexes of the model to detect apple leaf diseases. The results were assessed as true positive (TP), true negative (TN), false positive (FP), and false negative (FN).

Precision (accuracy rate), is the proportion of correctly recognized object A to the number of all objects recognized as A in the recognition result. Mean average precision is the result of averaging the accuracy rates of all types (Eq (3)).

$$\begin{array}{c} \text{Precision}=\frac{TP}{TP+FP} \end{array}$$ (3)

Recall (recall ratio), indicates the proportion of the number of objects correctly identified as A in the recognition result to the number of all objects identified as A in the test set (Eq (4))

$$\begin{array}{c} \text{Recall}=\frac{TP}{TP+FN} \end{array}$$ (4)

The IoU refers to the degree of overlap between the prediction box of object A and the original label box (Eq (5)).

$$\begin{array}{c} \text{IoU}=\frac{TP}{TP+FP+FN} \end{array}$$ (5)

Frame Rate per Second (FPS) is a common indicator of speed, namely, the number of pictures that can be processed per second.

### 3.2 Experimental results and analysis

**Fig 6:**
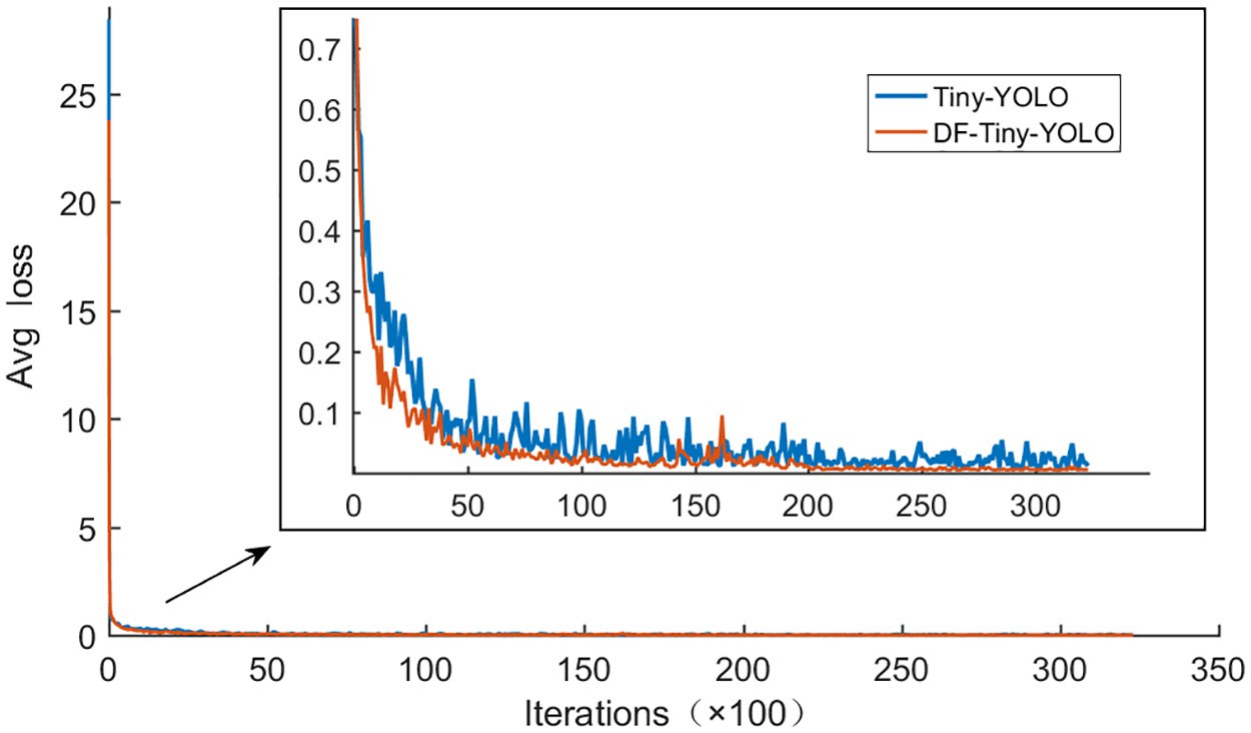
Average loss changes in DF-Tiny-YOLO and Tiny-YOLO. The figure shows variations in the average losses of DF-Tiny-YOLO and Tiny-YOLO during training.

To prevent the prediction model from overfitting, we determine whether the training model is optimal by observing the average loss value. We end the training when the average loss value decreases to a certain level and the value does not decrease again in the subsequent iterations. The training is stopped when the average loss of the network model decreases to the level of 0.0048. Fig 6 shows variations in the average losses of DF-Tiny-YOLO and Tiny-YOLO during training. Generally, the model rapidly fits at the start of the iteration, and the loss also rapidly decreases. With the continuous increase in the number of iterations, the loss gradually tended to stabilize during forward and backward oscillation. The loss values of DF-Tiny-YOLO and Tiny-YOLO in the first iteration were 23.79% and 28.46%, respectively. As the number of iterations increased, the loss values decreased faster in DF-Tiny-YOLO than Tiny-YOLO. The model fitting was faster, the oscillation amplitude is more moderate, and the final stationary value was relatively smaller. These results show that the proposed DF-Tiny-YOLO model has better training effects.

Table 5 compares the experimental results of DF-Tiny-YOLO, YOLOv2, and Tiny-YOLO. Compared with YOLOV2 and the original Tiny-YOLO, the detection effect of the DF-Tiny-YOLO model was somewhat improved. The mAP of DF-Tiny-YOLO is 99.99%, which was 0.17% higher than that of Tiny-YOLO. The average IoU was 90.88%, which was 1.59% and 10.38% higher than YOLOV2 and Tiny-YOLO, respectively. Recall was 1.00, which was the same as YOLOV2 and 0.01 higher than Tiny-YOLO. The detection speed was 280 FPS, which was the same as that of Tiny-YOLO, and the number of images that can be processed per second was 3-fold that of YOLOv2. Standard deviations and confidence intervals for each indicator are also shown here.

**Table 5:** Comparison of experiment results.

Training Network	Variable	Mean	Std. Err.	95% Conf. Interval
**DF-Tiny-YOLO**	mAP	99.99%	0.01	[99.97, 100.00]
	Average IoU	90.88%	0.12	[90.62, 91.14]
	Recall	1.00	0.00	[1.00, 1.00]
	FPS	280		
**YOLOv2**	mAP	99.99%	0.01	[99.98, 100.00]
	Average IoU	89.29%	0.49	[88.19, 90.39]
	Recall	1.00	0.00	[0.99, 1.00]
	FPS	93		
**Tiny-YOLO**	mAP	99.81%	0.03	[99.74, 99.88]
	Average IoU	80.50%	1.78	[76.47, 84.53]
	Recall	0.99	0.00	[0.98, 0.99]
	FPS	280		

The confusion matrix is a standard format for representing accuracy evaluation and is represented in this paper in the form of a matrix with 4 rows and 4 columns. In this experiment, the total number of each row of the confusion matrix represents the number of leaves predicted to be in that category, while each column represents the true attribution category of apple leaves. Table 6 shows the confusion matrix of DF-Tiny-YOLO, including the classification precision and Recall of four different classes of diseases. The matrix results reveal that the model mAP is 99.99% and the average IoU is 90.88%, indicating that the majority of the prediction results are consistent with the true situation and the model performs best for Apple cedar rust and healthy leaves.

**Table 6:** DF-Tiny-YOLO confusion matrix.

Classes	Apple Scab	Black Rot	Cedar Apple Rust	Healthy	Precision%	Recall
**Apple Scab**	299	1	0	0	99.99	1.00
**Black Rot**	1	299	0	0	99.97	1.00
**Cedar Apple Rust**	0	0	275	0	100.00	1.00
**Healthy**	0	0	0	529	100.00	1.00
**mAP (%)**	99.99%					
**Average IoU (%)**	90.88%					

The table shows the confusion matrix of DF-Tiny-YOLO, including the classification precision and Recall of four different classes of diseases.

**Fig 7:**
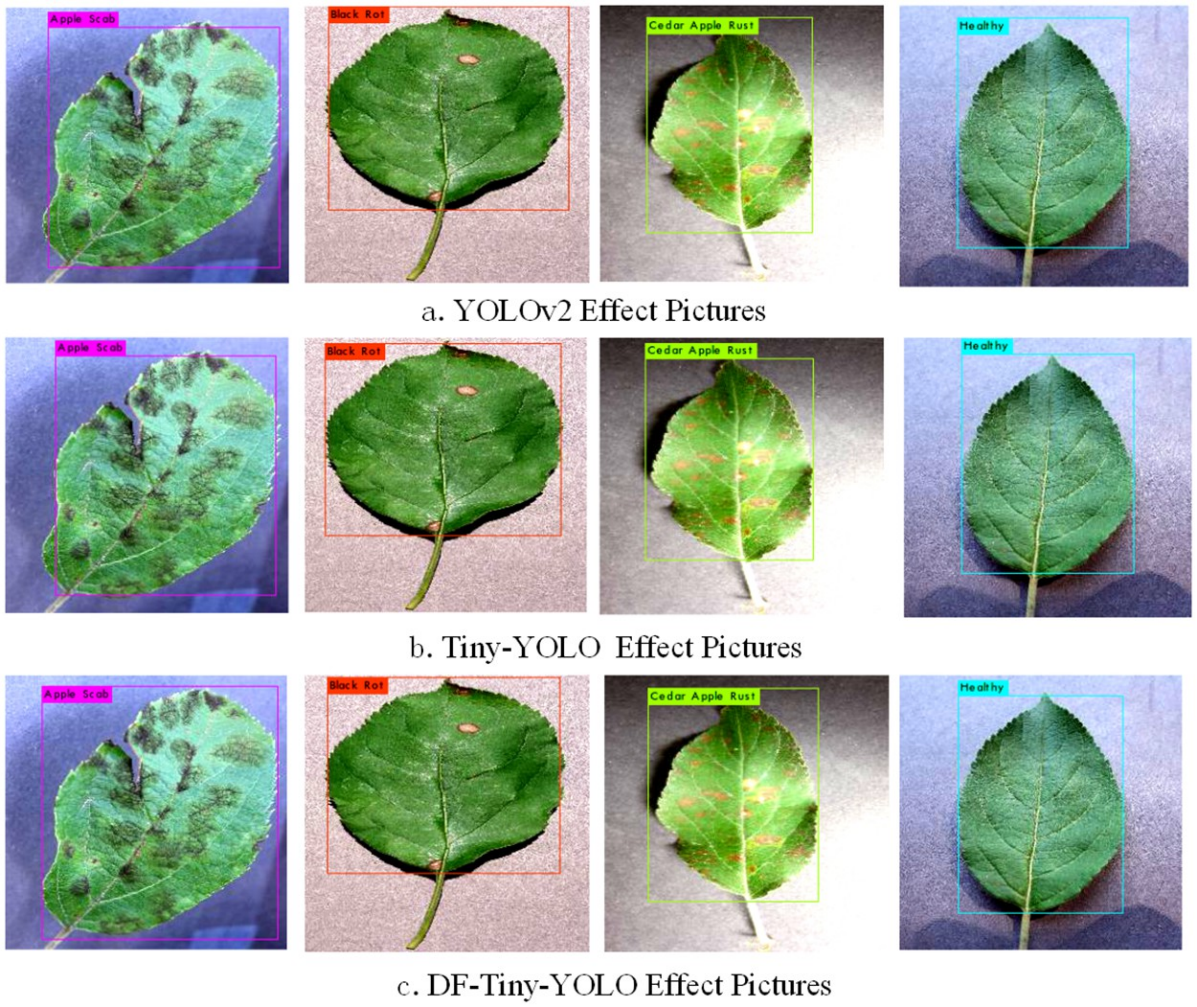
Disease detection by YOLOv2, Tiny-YOLO, and DF-Tiny-YOLO. The figures show the classification and localization effects of different models.

Fig 7 shows the detection of apple leaf diseases by DF-Tiny-YOLO, YOLOv2, and Tiny-YOLO. Based on output image results, each network model might have a specific degree of deviation, but the positioning box of DF-Tiny-YOLO was more in line with the target sample, the results were more inclusive, and the error rate was lower. This further showed that the proposed network model has specific advantages.

## 4. Conclusion

### 4.1 Research conclusion

Nowadays, much progress has been made in the detection of apple leaf diseases. When the traditional approach seemed to have hit a bottleneck, higher accuracy can be realized more easily using a deep learning approach based on convolutional neural networks. Especially, since target detection technology appeared during the 1960s, target detection based on deep learning has emerged, and convolutional neural network learning methods are now being applied to classify and identify apple leaf diseases, such as the SDD model, and the R-CNN and YOLO series of algorithms. Many improved schemes have been proposed based on existing algorithms.

Redmon, Divvala [38] launched the YOLOv1 target detection network in 2016 and it has since been improved in versions v2 and v3. The Tiny-YOLO model reduces the model parameter setting and accelerates the model training process. The present study improved the Tiny-YOLO model by combining DenseNet and F-YOLO network concepts and we propose that the DF-Tiny-YOLO apple leaf disease detection model can solve the problem of differentiating apple scab, black rot, and cedar rust from healthy apple leaves. These diseases are complicated, difficult to identify and have a low recognition rate among similar diseases. Our experimental results showed that the mAP of DF-Tiny-YOLO was 99.99%, the average IoU was 90.88%, and the detection speed was 280 FPS. Compared with previous studies done by scholars for apple leaf disease identification [29–32], DF-Tiny-YOLO has three major innovation: Firstly, this paper proposed a more advanced method for identifying the complex texture of apple leaves and differentiating similar diseases, which helps growers to accurately judge and rapidly deal with diseases, which further benefits growers. Secondly, in terms of the model structure, based on the model, This study optimized the structure of the original YOLO convolutional neural network model at three levels, and proposed the new DF-Tiny-YOLO network model, which detected apple leaf disease faster and more accurately. Thirdly, the results of comparative experimental exploration showed that among YOLOv2, Tiny-YOLO, the optimized DF-Tiny-YOLO network offered more advantages than the other two models. Therefore, we believe that the DF-Tiny-YOLO model can detect apple leaf diseases quickly and effectively.

Upgraded, more complex versions of the YOLO network algorithm have been developed. However, we selected Tiny-YOLO as the basic network for improvement because the model is simplified and fewer parameters need to be trained. Together with the combination of dense network and F-YOLO, the network model has further acquired more advantages based on maintaining its own advantages, which is an interesting innovation. The present study on the one hand, proved the effectiveness of our experimental enhancements and provided some experience with the development of current methods of apple leaf disease detection. On the other hand, more effective experiments can be conducted on this basis in the future.

### 4.2 Future research

Due to time, environmental and other constraints, there are still several areas for improvement and further expansion of the research in this paper.

1. Data availability is a prerequisite for research, and in apple leaf disease detection research, sufficient and diverse raw training data can help the effective training of detection models. At present, there are limited image data available on the Internet, and cooperation with apple plantations and agricultural research institutions is a useful and effective option.
2. Various new convolutional neural network models are emerging and existing models are being improved and developed, and more target detection models suitable for apple leaf disease detection are yet to be explored. However, the study is limited by the experimental environment, which makes it difficult to compare with more models. The future research work will focus on other excellent detection models and gradually optimize the results of apple leaf disease detection by using appropriate data enhancement techniques and network structure improvement methods.
3. In addition to apple leaf disease images, videos can also be used as detection objects, and real-time detection of more types of apple leaves during video shooting, etc. will also be the next step for effective implementation in subsequent research. With the deep learning technology in the field of target detection, several detection techniques have been effectively integrated, more and more new theories and methods have been proposed, and the accuracy and real-time performance of target detection have been improved, which will further help the research of apple leaf disease detection based on convolutional neural network.

## Supporting information

S1 File Experimental results. The file shows detailed results of experiments.
(XLSX)

S2 File (ZIP)
